# Analysis of clinical and radiological outcomes of long tibial stemmed total knee arthroplasty in knee osteoarthritis complicated by tibial stress fracture

**DOI:** 10.1186/s43019-022-00139-1

**Published:** 2022-02-22

**Authors:** Neelam V. Ramana Reddy, Mukesh Kumar Saini, Pera Jayavardhan Reddy, Ajay Singh Thakur, Challa Dinesh Reddy

**Affiliations:** Arthroplasty Division, Department of Orthopaedics, Star Hospitals, B Block Road No. 10 Banjara Hills, Hyderabad, India

**Keywords:** Stress fracture, Knee arthroplasty, Tibial extension, Fluted rods, Modular extenders

## Abstract

**Objective:**

Knee arthritis associated with tibial stress fractures represents an uncommon and difficult clinical scenario to treat. The use of long, fluted tibial extension rods has been vital in the management of such cases owing to immediate fracture stability and single-stage surgery without the need to open the fracture site. This study investigates clinical and radiological outcomes following total knee arthroplasty using a tibial extension stem in cases of knee osteoarthritis with tibial stress fracture.

**Methods:**

From February 2015 to December 2020, 17 patients who had total knee arthroplasty implanted with a long stemmed tibial component were included in the study. Patient data were analyzed for knee range of motion, deformities, Knee Society score, knee function score, and time to fracture union in the pre- and postoperative periods.

**Results:**

The mean follow-up duration was 22.7 ± 11.68 months (range 12–60 months), and mean time to fracture healing was 10.23 ± 2.81 weeks (range 8–20 weeks). The preoperative mean fixed flexion deformity improved from 8.53 ± 3.43° to a mean of 0.29°, and knee flexion improved from 79.4 ± 13.90° to 125.29 ± 8.74° on postoperative assessment. The Knee Society score improved from a mean preoperative score of 18.94 ± 5.55 (range 8–28) to 89.41 ± 7.5 (range 74–102, *p *value < 0.001). Similarly, the knee function score improved significantly from a mean preoperative score of 15.5 ± 4.48 (range 8–26) to a mean of 85 ± 6.09 (range 72–94, *p *value < 0.001).

**Conclusion:**

Total knee arthroplasty using long tibial extenders has been an effective and safe surgical option for patients with advanced osteoarthritis with tibial stress fractures.

## Introduction

Stress fracture is a failure of bony architecture to withstand repetitive, subthreshold stress, leading to partial or complete fracture that is not associated with any definite episode of energy trauma. It is further classified into insufficiency fractures and fatigue fractures, based on the characteristics of bony architecture [1]. While fatigue fracture occurs in normal bone subjected to abnormal high stresses over time, insufficiency fracture occurs with normal stress in already compromised bone due to certain conditions such as osteoporosis, osteomalacia, Paget’s disease, hyperparathyroidism, rheumatoid arthritis, and metabolic bone disease [1, 2].

Stress fracture elsewhere in the body are usually treated by rest, casting, and sometime surgery with internal fixation [1–3]. Stress fracture of proximal tibia in the setting of end-stage severe arthritis of the knee, occur secondary to severe malalignment in the coronal plane, leading to a concentration of stresses on the medial/lateral side of proximal tibia in varus/valgus knee [4, 5]. The stress concentration leads to partial or complete fracture of the bone at point of fatigue. This scenario presents a complex clinical condition to treat because of poor bone quality, elderly patient population, obesity, preexisting rheumatoid arthritis or steroid treatment, abnormal metabolic profile, and limited available evidence regarding the effectiveness of available treatment options.

Historic treatment options include nonoperative treatment in a cast, while operative treatment includes two stage surgery with internal fixation with or without osteotomy in the first stage and total knee arthroplasty (TKA) in the second stage, simultaneous total knee arthroplasty and internal fixation in one sitting, and simultaneous total knee arthroplasty with fracture fixation using tibial stem extensions [6, 7].

We describe an observational study of a series of 17 cases with end-stage knee arthritis associated with stress fractures of the proximal third of tibia treated with simultaneous total knee arthroplasty using long, fluted stemmed tibial components to ensure rotational stability distal to the fracture site.

## Patients and methods

### Study population and data collection

Patients with end-stage degenerative knee arthritis associated with stress fracture of the proximal third of the tibia treated at our institute from February 2015 to December 2020 were analyzed.

**Fig. 1:**
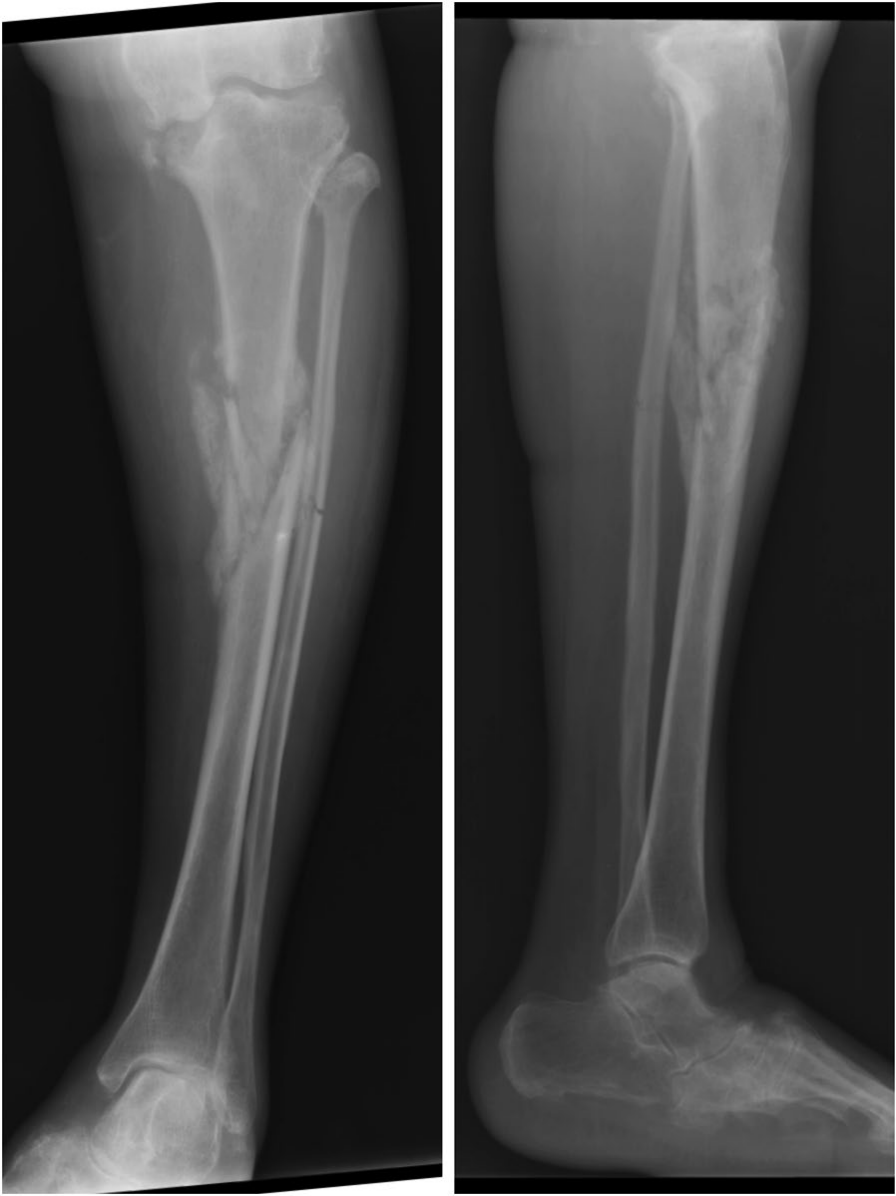
Case 1 preoperative radiographs depicting grade 4 osteoarthritis of the knee with stress fractures (both extraarticular and intraarticular). The patient had history of long standing knee pain since many years, with inability to walk since the last 4 months. The tibial alignment appears to be in severe varus (> 20°)

**Fig. 2:**
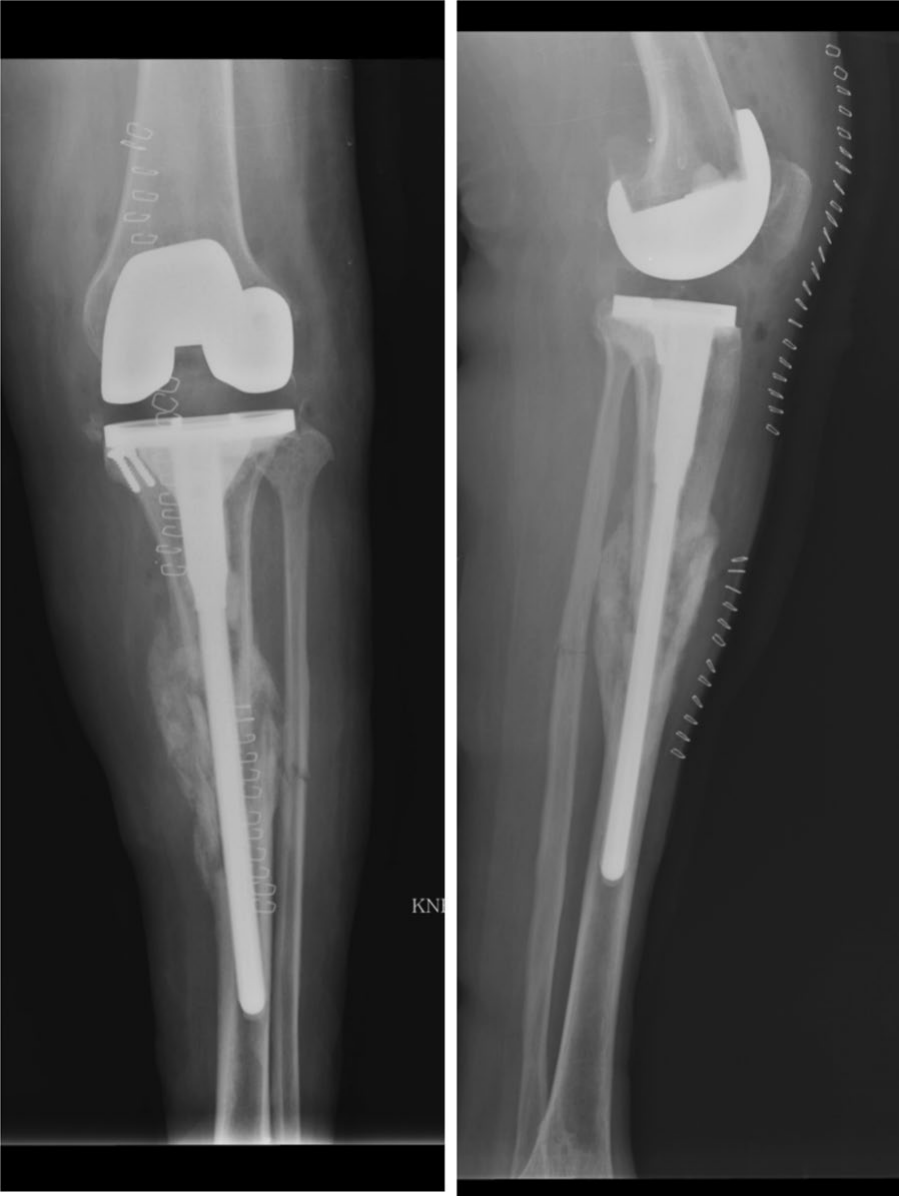
Postoperative radiograph of case 1 patient showing total knee arthroplasty components with long tibial extension rod. The medial plateau fragment was excised and the resultant contained defect was built up with two cortical screws and bone cement. Although the overall postoperative alignment remained as varus, the patient had uneventful recovery during the postoperative period and fracture healing

**Fig. 3:**
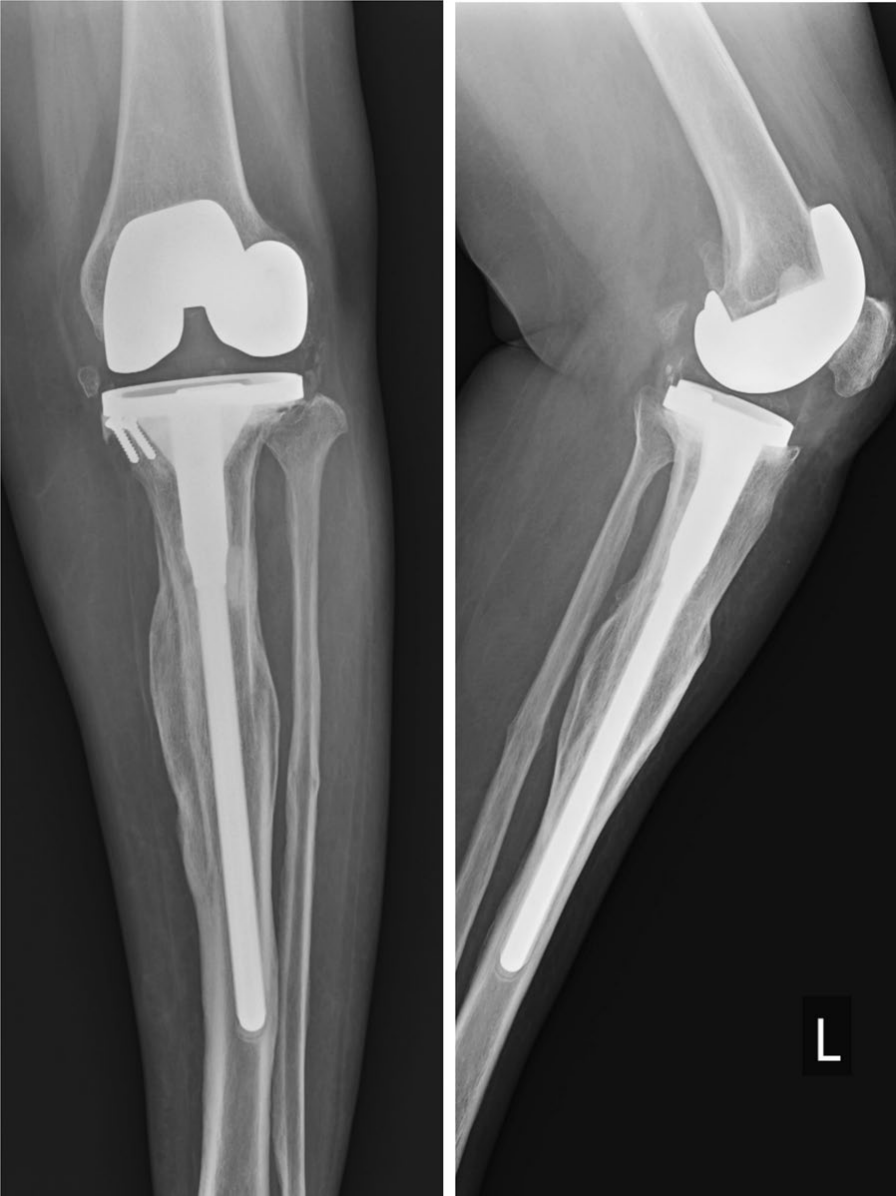
Radiograph of the case 1 patient at 5-year follow-up showing solid union of extraarticular fracture. The patient did not have any complaints, though a radiolucent line is seen just below the lateral tibial plate

**Fig. 4:**
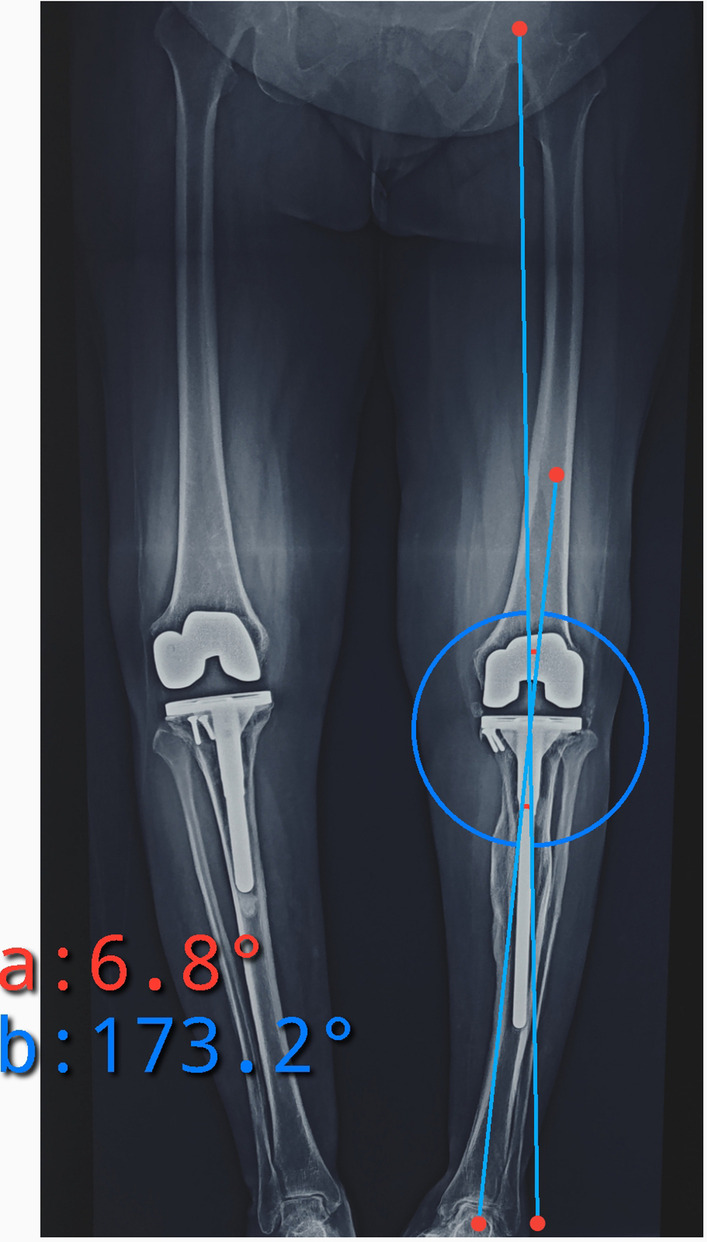
Scanogram involving both lower limbs and pelvis of the case 1 patient shown in Figs. 1, 2, 3 depicting varus malunion of the tibial stress fracture and resulting mechanical axis deviation into varus (6.8°). Despite the malalignment, the patient remained asymptomatic with good function

All patients had varus deformity and only extraarticular stress fracture of the tibia, except one case that had both extraarticular and intraarticular fractures of medial tibial condyle (Figs. 1, 2, 3). This patient had severe varus deformity (> 20°) in the preoperative period, which could be corrected to 6.8° hip–knee–ankle (HKA) angle in postoperative radiograph (Fig. 4). Despite having varus malalignment, this patient had an uneventful recovery, including fracture union, and remained asymptomatic with decent function [Knee Society score (KSS) = 89 and knee function score (KFS) = 90] at 5 years of follow-up. All cases were treated by single-stage TKA using a tibial component with fluted tibial extension rod.

### Preoperative assessment

**Fig. 5:**
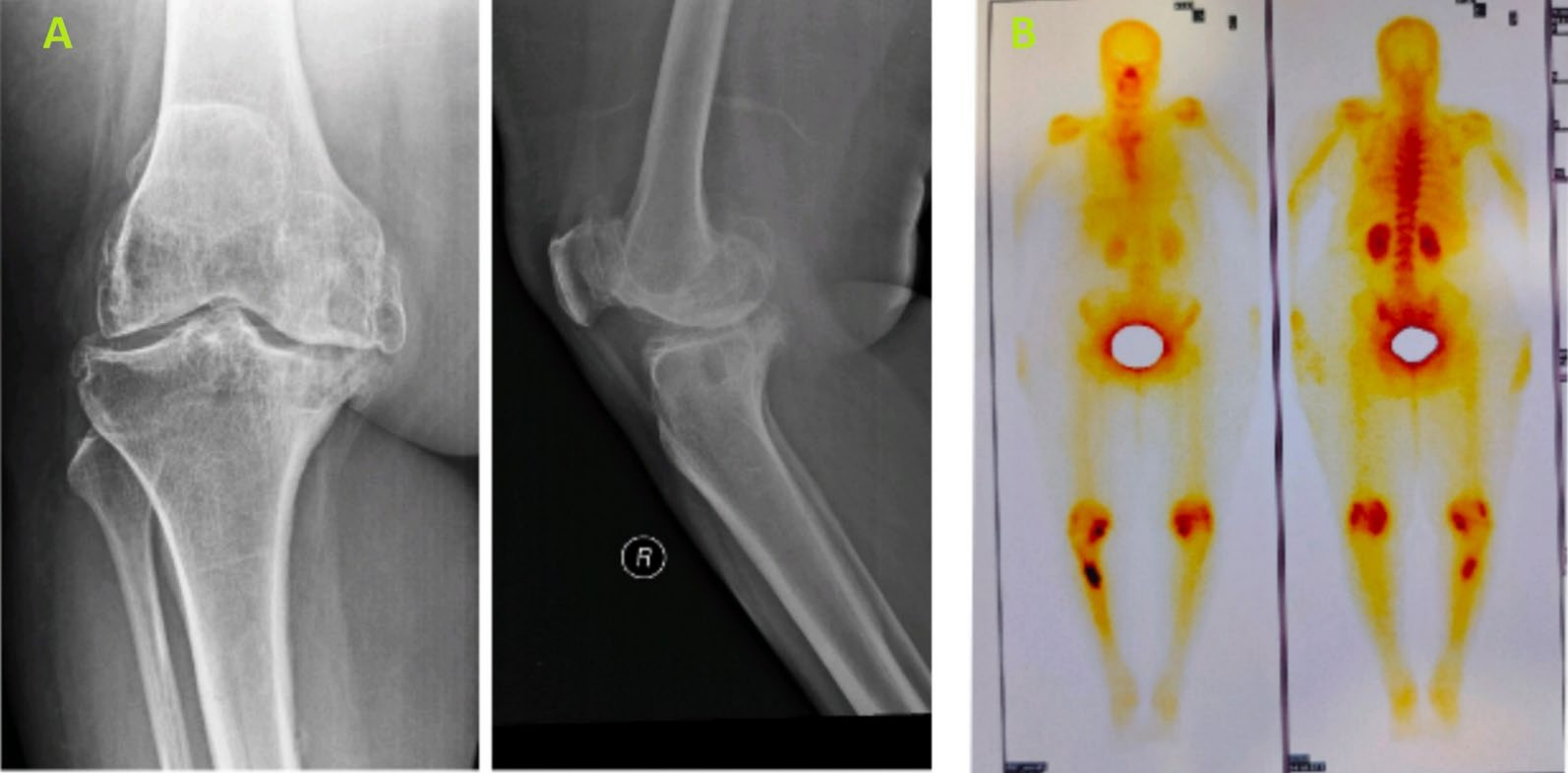
**A** On the left, preoperative radiograph of case 2 showing grade 4 osteoarthritis of the knee. The patient also had shin tenderness but no clear fracture line on the radiographs. **B** On the right, nuclear bone scan of the case 2 patient showing diffuse extensive lesion in right tibia with highly increased activity in the corticomedullary region

**Fig. 6:**
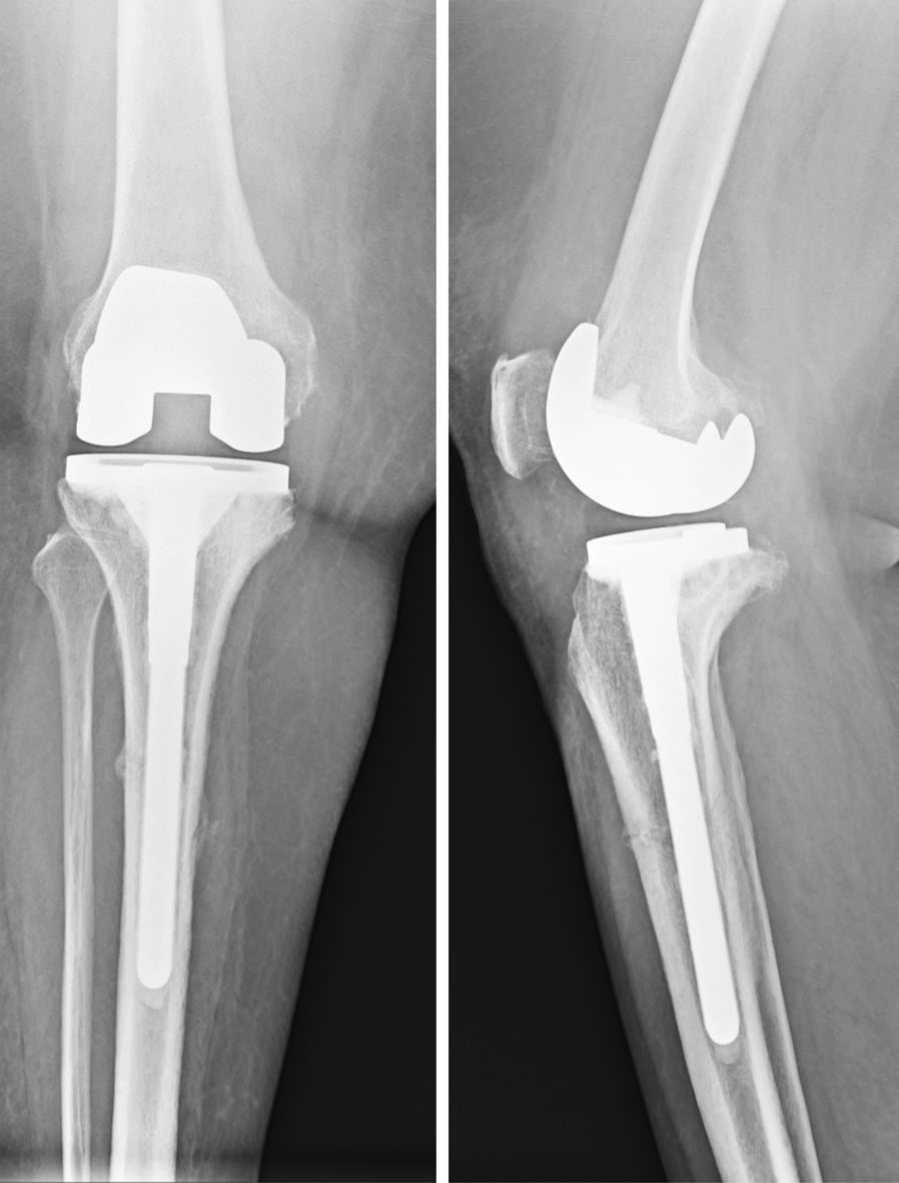
Postoperative 12 month follow-up radiographs of case 2 operated with total knee replacement using long stem extender on tibial side. Notable fracture healing is evident by periosteal new bone formation

The patients were evaluated preoperatively for knee range of motion (ROM), fixed deformities, knee society score (KSS), knee function score (KFS), mechanical tibio–femoral angle calculated on long leg standing radiographs, standard antero–posterior (AP), lateral, and skyline radiographs. While most of the stress fractures were diagnosed on plain X-rays, two patients needed nuclear bone scans to confirm the diagnosis (Figs. 5, 6).

X-ray findings of definite fracture line, periosteal or endosteal bone formation, or linear sclerosis were taken as an evidence of stress fracture. Preoperative metabolic workup in the form of serum calcium, phosphorus, vitamin D, alkaline phosphatase (ALP), and parathyroid hormone (PTH) levels were done.

### Operative technique and follow-up

All patients underwent cemented total knee arthroplasty under spinal anesthesia using posterior cruciate sacrificing (PS) implants (Press Fit Condylar Sigma, DePuy Johnson & Johnson, New Brunswick, USA) with modular fluted tibial extension rods. An inflatable tourniquet was used in all cases from skin incision to closure, set at 100 mm Hg above the systolic blood pressure of the patient. A standard medial parapatellar arthrotomy approach was used in all cases. After preparing the femoral side with standard measured resection technique, a tibial cut was done using an extramedullary jig and sequential intramedullary reaming done after correction of any deformity at the fracture site.

A fluted extension rod acting as an internal splint provided excellent intraoperative stability at the fracture site, achieving adequate press fit in the distal tibial fragment ensuring rotational stability. The length of the rod was dictated by the location of the fracture as it was supposed to bypass the fracture site by at least two cortical diameters distally. Only the tibial base plate was cemented, taking care to prevent leakage of cement at the fracture site. Drains were not used.

In the postoperative period, all patients were encouraged to walk at full weight-bearing using a walker frame, starting from the first postoperative day. Knee range of motion (ROM) and dynamic quadriceps and hamstring exercises were started from the first postoperative day and escalated depending on the pain tolerance of the patient. The walker frame was removed after 3 weeks, and the patients were asked to switch to elbow crutches or sticks for the next 3 weeks, which they were also weaned off ultimately. Patients were followed-up with clinical and radiological assessments at 8, 10, and 12 weeks, 6 months, and annually thereafter. Radiological union (at least three cortices with bridging callus) with absence of pain and tenderness at fracture site was defined as union of fracture.

Functional outcome assessment was done by recording data on postoperative knee range of motion, Knee Society score, knee functional score, and radiological evaluation using standard AP and lateral projections of the knee. The postoperative tibial alignment was also evaluated using the anatomical axis of the tibia to assess the adequacy of fracture reduction.

### Statistical analysis

The statistical analysis was done using SPSS for windows version 23. Descriptive data were presented as mean for continuous variables. To find out significant differences between two dependent variables, a paired *t*-test was used to compare the means, e.g., for Knee Society score and knee function score. A two-sided *p *value < 0.05 taken as statistically significant.

## Results

Demographic data showed the mean age to be 65.35 ± 4.88 (range 58–72) years (Table 1). Out of 17 patients, 13 were females and only 4 were males. The preoperative mean body mass index (BMI) was 32.4 ± 2.48 (range 28–37) kg/m^2^.

**Table 1:** Patient characteristics

Characteristic	Value
Age (years)	65.35 ± 4.88
Sex	Female = 13, Male = 4
Body mass index (kg/m^2^)	32.4 ± 2.48
HKA angle (degrees)	21.20 ± 8.4 varus
Follow-up period (months)	22.7 ± 11.68
Time to fracture union (weeks)	10.23 ± 2.81

The preoperative mean varus deformity, measured as the hip–knee–ankle (HKA) angle was 21.20 ± 8.4° (range 10–35°) of varus, while the mean HKA angle in postoperative patients was 1.22 ± 1.6° of varus alignment. Most patients had postoperative alignment of HKA angle < 3°, except the one case depicted in Figs. 3, 4, which had 6.8° varus and, as described earlier, had good outcome. The postoperative tibial anatomical alignment was also evaluated to assess the fracture reduction. Since most of our patients had chronic tibia stress fractures that remained undisplaced due to undisturbed periosteum and intact fibula, gross displacement was not seen preoperatively. In the postoperative period, the coronal alignment was checked using the anatomical tibia axis, which remained within 5° varus/valgus (except the one case illustrated in Fig. 3). The case illustrated in Fig. 3 had gross varus displacement (which was correctable to neutral) and mobility in coronal and sagittal planes. However, during intraoperative evaluation while trial component testing, we felt that the overall alignment was around 3° of varus, hence, we decided against fibular osteotomy.

The preoperative mean fixed flexion deformity was 8.53 ± 3.43° (range 5–15°), which improved to 0.29° (only one patient had 5° of fixed flexion deformity while the other patients had complete extension). The mean preoperative flexion angle was 79.41 ± 13.90° (range 60–100°), improved to 125.29 ± 8.74° (range 110–140°) on postoperative assessment (Table 2). Most of the patients had normal metabolic profile except hypovitaminosis D detected in 12 patients (four patients had mild deficiency with levels in range of 15–20 ng/mL and eight patients had severe deficiency with levels less than 5 ng/mL). Fifteen patients had elevated serum ALP levels (all > 500 IU/L) and two patients had normal levels (reference 40–150 IU/L).

**Table 2:** Clinical and radiological outcome parameters compared pre- and postoperatively

Outcome parameter	Preoperative value	Postoperative value	*p *-Value
Fixed flexion deformity (degrees)	8.53 ± 3.43	0.29 ± 1.21	< 0.001
Flexion angle (degrees)	79.41 ± 13.90	125.29 ± 8.74	< 0.001
Knee society score	18.94 ± 5.55	89.41 ± 7.5	< 0.001
Knee function score	15.5 ± 4.48	85 ± 6.09	< 0.001

Mean follow-up duration was 22.7 ± 11.68 months (range 12–60 months) and mean time to fracture healing was 10.23 ± 2.81 weeks (range 8–20 weeks). The Knee Society score improved from a mean preoperative score of 18.94 ± 5.55 (range 8–28) to 89.41 ± 7.5 (range 74–102, *p * < 0.001). Similarly, the knee function score improved significantly from a mean preoperative score of 15.5 ± 4.48 (range 8–26) to 85 ± 6.09 (range 72–94, *p * < 0.001).

All fractures got united uneventfully, and no patient had any complication in terms of wound healing, nonunion, joint instability, or patellar complications.

## Discussion

Stress fracture associated with end-stage knee arthritis is relatively uncommon and underreported, especially in developing countries due to ignorance of pain, financial constraints, and misplaced fear of surgical procedures, hence patients tend to report later in the disease process when they become bedridden or wheelchair bound [8]. The purpose of this study was to review the clinical and radiological outcomes following the use of fluted, long uncemented tibial stem extenders with knee arthroplasty to alleviate patient symptoms, correct the deformity, and provide a stable painless mobile knee joint with a single surgical procedure. We conclude the results in terms of knee function, fracture union, and postoperative alignment of the limb with short-term survival data of the prosthesis. The coronal plane deformity shifts the mechanical axis of the lower limb, leading to a concentration of stresses and subsequent failure of either of cortices of the tibia. Diagnosis was made with a typical history of shin pain, along with knee pain that is usually insidious onset, along with tenderness, joint crepitus, and typical deformity [3, 8].

Confirmation of the diagnosis is usually done on routine radiographs, but there are chances that the fracture line can be missed when it is distal to the meta-diaphyseal junction of the tibia, and it is recommended to have an index of clinical suspicion in those elderly patients who remain wheelchair bound and refuse to walk on outpatient clinical examination. Rarely, the very faint fracture line on routine radiographs can be missed, and in those cases, a nuclear bone scan is mandatory to confirm the diagnosis.

Routine biochemical tests should be performed to rule out metabolic disease. In our series, all patients had normal metabolic profile except elevated serum ALP (in 15 patients) and hypovitaminosis D (in 12 patients), which were treated with oral supplements of cholecalciferol.

Tibial stress fractures coexisting with knee arthritis were first described first by Wheeldon in 1961 [1]. Various subsequent reports were limited to a few cases only, and advocated conservative treatment as the modality of choice, in the form of rest, limited activities, and plaster application [2–4].

Other options proposed were internal fixation with or without osteotomy in the first stage and TKA in the second stage. The two-stage technique produced fair results but has been widely criticized due to unavoidable complications of immobilization such as knee stiffness and muscles wasting [5].

The goals of surgical treatment in such scenarios should be as follows:

1. Restoration of normal mechanical alignment of lower limb
2. Achieving fracture union by providing stable and internal fixation
3. Replacement of arthritic joint with prosthetic joint providing pain relief and commencement of mobilization

Restoration of mechanical axis of the knee, along with reduction and fixation of tibial stress fractures, poses an unfamiliar challenge. Due to a concentration of stress, the medial side (in varus deformity) or lateral side (in valgus deformity) acts as the compression side and the opposite cortex as the tension side. By restoring mechanical alignment of the lower limb (with TKA), the deforming forces are neutralized and converted to a vertical compression force across the fracture site by bridging the fracture and providing internal splinting with an extension stem. Treating only the fracture, without correction of alignment, may lead to malunion, nonunion, persistent knee pain, and recurrence of the stress fracture [9, 10]. Simultaneous TKA with fluted, press fit, long tibial extension rods has been previously advocated in few case series with good to excellent clinical outcomes [6–8, 11, 13, 15].

Ng and colleagues described two such cases of knee OA with proximal third tibial diaphysis stress fractures, which were initially treated conservatively. Subsequently, after failure of conservative treatment and fracture nonunion, knee arthroplasty with tibial stem extensions proved to be satisfactory treatment in their case studies [16].

The study by Mullaji and colleagues was the largest previous series published, including 34 patients [6]. The authors proposed a classification based on the site of stress fracture (intraarticular versus extraarticular) or chronicity of fracture (impending, acute, malunited, or ununited). The authors reported union in all cases without any complications using tibial stem extenders, except one case in which they observed cortical perforation by extension rod. However, the follow-up remained uneventful with good recovery. Based on these reviews and our observations, we can conclude that all extraarticular tibial stress fractures can be addressed with tibial stem extension of adequate fit and length. While with intraarticular rim fractures, the smaller plateau fragments can be excised safely (advantageous in cases of severe varus deformity), the larger fragments need protection with stem extenders or reconstruction of the resultant defect with cement, wedges, or augments [6]. Similarly, Savant et al. also reported satisfactory clinical outcomes in a series of four cases treated with total knee arthroplasty using a long tibial extension rod [7].

Dhillon et al. described a series of eight cases of osteoarthritis knee with extraarticular tibial stress fractures treated with stem extenders (in six patients), while the other two patients received an additional unicortical plate for rotational stability [8]. However, the author concluded that the chances of wound breakdown and infection increases significantly with the use of such plates. Moreover, the plate, by its load-sharing effect, prevents compressive forces at the fracture site, leading to a delay in union and hence its use should be avoided in such cases. The notion of deleterious effects of load sharing by the plate was also supported by Rashid et al. in their series. The authors summarized that all extraarticular stress fractures should be managed with press fit stem extenders after intramedullary reaming and without opening the fracture site [14].

In this study, a long, fluted titanium tibial stem extension was used, which ensured press fit at the distal fragment of the tibial diaphysis and provided enough rotational stability to allow immediate postoperative mobilization. Other studies have also reported excellent outcomes from using tibial stem extenders in knee osteoarthritis complicated with stress fracture of the tibia, without the need for additional internal fixation device [11–14]. In most cases of ununited chronic stress fractures of the tibia, the fracture remains stable and postoperative malalignment is not seen; however, in cases of mobile, displaced fractures, there is a tendency to develop varus deformity due to intact fibula (Fig. 3, Fig. 4). Therefore, we believe that addition of corrective tibial osteotomy, with or without fibular osteotomy should be reserved for the correction of residual varus to achieve neutral coronal tibial alignment in case of malunion or stiff fibrous union, as suggested in a few reports [6–8, 14].

Although the single stage surgical option seems to be very effective and safe, the surgeon must be careful while reaming for the stem insertion because of the possibility of cortical breach and malalignment due to under-corrected deformity at the fracture site, poor bone stock, and the potential for periprosthetic fractures. We did not encounter any such adverse event in our series, but it has been reported previously in literature [6].

Careful and good surgical planning remains vital to understand the bony morphology and anticipation of possible intraoperative complications. The strength of our study is the relatively long follow-up period, while the weakness are the small number of cases and retrospective nature of the study.

## Conclusion

We conclude that simultaneous TKA using long, fluted tibial extension rods is an effective and safe surgical option for patients with end-stage osteoarthritis associated with proximal tibial stress fractures. The advantages are that this is a single stage procedure without the need for additional incisions or internal fixation devices, there are no wound healing complications, and postoperative stability is immediate, leading to fully fledged mobilization of patients in the early postoperative period, thus minimizing fracture-related morbidity and complications.

## Data Availability

The authors declare no conflict regarding the data transparency.
